# Investigations on the substrate binding sites of hemolysin B, an ABC transporter, of a type 1 secretion system

**DOI:** 10.3389/fmicb.2022.1055032

**Published:** 2022-12-01

**Authors:** Zohreh Pourhassan N., Eymen Hachani, Olivia Spitz, Sander H. J. Smits, Lutz Schmitt

**Affiliations:** ^1^Institute of Biochemistry, Heinrich Heine University, Düsseldorf, Germany; ^2^Center for Structural Studies, Heinrich Heine University, Düsseldorf, Germany

**Keywords:** ABC transporter, protein secretion, putative binding pockets, substrate interaction, bacterial secretion systems

## Abstract

The ABC transporter hemolysin B (HlyB) is the key protein of the HlyA secretion system, a paradigm of type 1 secretion systems (T1SS). T1SS catalyze the one-step substrate transport across both membranes of Gram-negative bacteria. The HlyA T1SS is composed of the ABC transporter (HlyB), the membrane fusion protein (HlyD), and the outer membrane protein TolC. HlyA is a member of the RTX (repeats in toxins) family harboring GG repeats that bind Ca^2+^ in the C-terminus upstream of the secretion signal. Beside the GG repeats, the presence of an amphipathic helix (AH) in the C-terminus of HlyA is essential for secretion. Here, we propose that a consensus length between the GG repeats and the AH affects the secretion efficiency of the heterologous RTX secreted by the HlyA T1SS. Our *in silico* studies along with mutagenesis and biochemical analysis demonstrate that there are two binding pockets in the nucleotide binding domain of HlyB for HlyA. The distances between the domains of HlyB implied to interact with HlyA indicated that simultaneous binding of the substrate to both cytosolic domains of HlyB, the NBD and CLD, is possible and required for efficient substrate secretion.

## Introduction

Gram-negative bacteria have evolved type 1 secretion systems (T1SS) to translocate a diverse variety of substrates, mainly virulence factors, in one step to the exterior space (Holland et al., 2016). The substrates of T1SS are transported in an unfolded state and classified based on their functionality, as follows: toxins, lipases, heme-binding proteins, adhesion proteins, proteases, S-layer binding proteins, and many of unknown functions (Linhartová et al., 2010; Thomas et al., 2014).

In terms of architecture, T1SS nanomachineries consist of three components: an ATP binding cassette (ABC) transporter, a membrane fusion protein (MFP), and an outer membrane protein (OMP). These three components form a continuous channel, spanning both, inner and outer, membrane for redirecting substrates in one step from the cytosol to the extracellular space (Pourhassan et al., 2021).

A common feature of T1SS substrates is the presence of a secretion signal in the extreme C-terminus, which is not cleaved prior, while, or after secretion (Pourhassan et al., 2021). Additionally, these substrates harbor repetitive Glycine- and Aspartate-rich sequences, named as either nonapeptide sequence or GG repeats, having the consensus sequence GGxGxDxUx (x refers to any amino acid; U refers to a large and hydrophobic amino acid) upstream of the secretion signal (Bumba et al., 2016).

The GG repeats are characteristic for a group of T1SS substrates known as RTX (repeats in toxins) family, a superfamily of mostly toxic proteins secreted by Gram-negative bacteria. It has been demonstrated that the RTX is a site for binding of Ca^2+^ ions stimulating the folding of a substrate in the extracellular space where the Ca^2+^ concentration (around 2 mM) is higher than the K_D_ of the RTX (around 150 μM) to Ca^2+^. Obviously, substrates remain unfolded in the cytosol where the Ca^2+^ concentration is only around 300 nM (Bumba et al., 2016; Spitz et al., 2022).

The HlyA secretion system is a paradigm of the T1SS, first identified in an uropathogenic *Escherichia coli* strain (Noegel et al., 1979). The secretion machinery of this system consists of three membrane components, as follows: the ABC transporter HlyB, the membrane fusion protein HlyD, and the outer membrane TolC (Holland et al., 2016; Pourhassan et al., 2022). Only very recently, structural information derived from single particle cryo-EM was reported and revealed an unusual six stoichiometry of the inner membrane components of the HlyA T1SS, HlyB, and HlyD, which form the so-called inner membrane complex (Zhao et al., 2022). This structure revealed that a trimer of HlyB dimers incorporated with in sum six HlyD monomers. This complex formation of HlyB and HlyD is essential for secretion. They also reported a novel organization of HlyB trimers in which three perform ATP hydrolysis, but only one acts as the protein channel for the HlyA secretion (Zhao et al., 2022).

Another protein of this system is the acyl carrier HlyC that acylates two specific lysin residues of HlyA and converts it from an inactive form into an active toxin. However, this posttranslational modification is not required for secretion (Hardie et al., 1991).

HlyA is a member of RTX family with a size of 110 kDa and has the ability to create a pore in the membrane of, for example, human erythrocytes. The required information for the secretion of HlyA is encoded within the last 50–60C-terminal amino acids (Nicaud et al., 1986). A C-terminal fragment of HlyA, termed HlyA1, has been used frequently as a transport-carrier for secretion of different heterologous proteins. HlyA1 consists of the C-terminal secretion signal along with three of the six conserved GG repeats (Pourhassan et al., 2021).

Extensive research on the T1SS substrates demonstrated that a secondary structure might be encoded by the secretion signals, since no significant conservation on the primary structure of the secretion sequences was evident. In this regard, the presence of an amphipathic helix (AH) located between residues 973 and 987 of HlyA was proposed (Koronakis et al., 1989), and only recently further data was provided by Spitz et al. supporting the idea that the presence of this AH in the C-terminus of HlyA plays an essential role in the early steps of the secretion process (Spitz et al., 2022). In the current study, we identified a consensus length between the AH and the GG repeats that influences the secretion efficiency of heterologous RTX proteins secreted by the HlyA T1SS. We observed that a reduction in the secretion rate of HlyA occurs by shortening this consensus length.

It is now understood that secretion through HlyA T1SS occurs within an ordered reaction. This reaction initiates upon interaction of HlyA with the inner membrane complex, consisting of HlyB and HlyD. This interaction recruits TolC protein, and only then all these membrane proteins form a channel bypassing HlyA in one step, the C-terminus first (Lenders et al., 2015), to the extracellular space (Letoffe et al., 1996; Thanabalu et al., 1998).

The central protein of the HlyA T1SS is the ABC transporter HlyB. In terms of topology, HlyB harbors six transmembrane domains (TMD), a nucleotide-binding domain (NBD), and a C39-peptidase-like domain (CLD) (Holland et al., 2016). Concerning the CLD, despite 40% homology to other C39-peptidases, no protease activity has been reported for this domain, because of a defective catalytic site. Nevertheless, it is essential for the secretion. Importantly, Lecher et al. reported an interaction between the CLD and the unfolded substrate outside of the secretion signal, suggesting a receptor or a chaperone-like activity for this domain, but the exact function of this domain remains unclear (Lecher et al., 2012).

The NBD domain not only provides the energy of the transport by hydrolyzing ATP (Schmitt et al., 2003; Zhao et al., 2022), but also interacts with HlyA prior to the secretion, revealed by surface plasmon resonance analysis (Benabdelhak et al., 2003). This interaction vanishes in the absence of the signal sequence of HlyA, and is highly dependent on the presence of nucleotides either ATP or ADP (Benabdelhak et al., 2003). Like other ABC transporters, the ATP binding site of HlyB is highly conserved and sequence analysis reveals the presence of three characteristic motifs, the Walker A motif with the sequence of GXXGXGKS/T (x refers to any amino acid), the Walker B motif with the sequence of Φ Φ Φ Φ D (Φ refers to any hydrophobic amino acid), and the C-loop with the sequence of LSGGQ (also called as signature motif) (Oswald et al., 2006).

Lecher et al. already reported the substrate binding region within the CLD by conducting structural studies and pulldown assays (Lecher et al., 2012). Regarding the binding sites of NBD for HlyA no study has been conducted so far. Here, we were aiming to address this question by performing *in silico* studies. Furthermore, we analyzed the available structures of the NBD of HlyB and found two possible interaction sites for the AH of HlyA. Our mutagenesis and functional studies also support the presence of these two interaction sites. In the following, these interaction sites as well as the previously identified HlyA interaction site of the CLD were mapped onto a model of HlyB suggesting that simultaneous binding of the substrate to both cytosolic domains of the transporter is necessary for efficient substrate secretion.

## Materials and methods

All chemicals used in this study were purchased from Sigma-Aldrich, Applichem GmbH, and Roche Diagnostics GmbH if not stated otherwise. Enzymes were purchased from New England Biolabs. All oligonucleotides were purchased from Eurofins MWG Operon and diluted in MilliQ-water. The NucleoSpin plasmid miniprep kit and the Q5 site-directed mutagenesis kit were purchased from Macherey Nagel (Dueren, Germany) and New England Biolabs, respectively. DNA sequencing was performed at Microsynth Seqlab (Göttingen, Germany).

### Construction of plasmids used in this study

In this study *E. coli* DH5α, *E. coli* BL21-Gold (DE3), and *E. coli* BL21 (DE3) were used for cloning, overexpression, and purification purposes. Plasmids and oligonucleotides used in this study are listed in Tables 1, 2, respectively.

**Table 1:** Plasmids used in this study.

Plasmids name	Source
pK184_*hly*BD	Khosa et al. (2018)
pK184_*hly*B (F518D, Y519D)-*hly*D	This study
pK184_*hly*B (Y477D, F518D, Y519D)-*hly*D	This study
pK184_*hly*B (V682D)-*hly*D	This study
pK184_*hly*B (V682D, L697D, Y700D)-*hly*D	This study
pPSG122_His::NBD	Zaitseva et al. (2004)
pPSG122_His::NBD (Y477D)	This study
pPSG122_His::NBD (F518D, Y519D)	This study
pPSG122_His::NBD (Y477D, F518D, Y519D)	This study
pPSG122_His::NBD (V682D)	This study
pPSG122_His::NBD (L697D, Y700D)	This study
pPSG122_His::NBD (V682D, L697D, Y700D)	This study
pSU2726_*hly*C (159 bp)-*hly*A	Khosa et al. (2018)
pSU2726_*hly*C (159 bp)-*hly*A (Δ939-968)	This study
pSU2726_*hly*C (159 bp)-*hly*A (Δ919-968)	This study

**Table 2:** Oligonucleotides used in this study.

Name	Details	Sequence
P1	Fp, pbp-in, Y477D	CCGGTTTCGCGATAAGCCTGACT
P2	Rp, pbp-in, Y477D	ATATTACGAAAAGTGATATCACCATTAATTTC
P2’	Rp, pbp-in, Y477D	ATATTACGAAAAGTGATATCGTG
P3	Fp, pbp-in, F518D, Y519D	AATTCAACGTGATGATATTCCTGAAAATGGC
P4	Rp, pbp-in, F518D, Y519D	AATTTAGTTAATGTGCTTTTTCC
P5	Fp, pbp-out, V682D	AGGGAAAATTGATGAACAGGGTAAAC
P6	Rp, pbp-out, V682D	TTTTCCATGACAATAATGCG
P7	Fp, pbp-out, L697D, Y700D	AGTGATTTATATCAGTTACAGTCAGAC
P8	Rp, pbp-out, L697D, Y700D	GTAATCACTTTCCGGTTCAGAAAG
P9	Fp, deletion of 30 or 50 residues of HlyA	AGTCAGGGTGATCTTAATCC
P10	Rp, deletion of 30 residues of HlyA	GATTATCCGGCCACTTTTATC
P11	Rp, deletion of 50 residues of HlyA	CTCTTTTTCAAACCAGTTCCTG

Fp, forward primer; Rp, reverse primer.

### Plasmids containing truncated variants of HlyA were constructed as follows

Plasmid pSU2726_*hlyC* (159 bp)-*hlyA* was used as the backbone plasmid. This plasmid contains an ampicillin resistance gene. The *lac* promoter of this plasmid is inducible with isopropyl β-D-1-thiogalactopyranoside (IPTG). To delete the encoding region of 30 and 50 residues of HlyA, plasmid pSU2726_*hlyC* (159 bp)-*hlyA* was linearized *via* PCR reaction using primer sets of P9/P10 and P9/P11, respectively. Subsequently, both PCR products were incubated with the KLD (kinase-ligase-DpnI) reaction mix of the Q5 site-directed mutagenesis kit (5 min, room temperature), according to the instructions of the manufacturer. Subsequently, 2 μL of the KLD mixture was transformed into chemically competent cells of *E. coli* DH5α. The sequence of the constructed plasmids pSU2726_*hlyC* (159 bp)-*hlyA* (Δ939-968) and pSU2726_*hlyC* (159 bp)-*hlyA* (Δ919-968) were confirmed *via* plasmid sequencing.

### Mutations in hlyB gene were introduced as follows

In general, plasmid pK184_*hlyBD* was used as the backbone plasmid. The *lac* promoter of plasmid pK184 is inducible with IPTG. This plasmid has a kanamycin resistance gene. The mutations were introduced in the *hlyB* gene using the Q5 site-directed mutagenesis kit, according to the instructions of the manufacturer. First the PCR for introducing each mutation was performed with the proper primer sets. Then, the PCR product was incubated with the KLD mix (5 min, room temperature). Subsequently, 2 μL of the KLD mixture was transformed into chemically competent cells of *E. coli* DH5α.

In detail, to introduce the mutations concerning the putative binding pocket inside (pbp-in), first plasmid pK184_*hlyB* (F518D, Y519D)-*hlyD* was constructed using primer sets of P3/P4. To introduce the third mutation of pbp-in, plasmid pK184_*hly*B (F518D, Y519D)-*hly*D was amplified using primer sets of P1/P2. The successful construction of plasmid pK184_*hlyB* (Y477D, F518D, Y519D)-*hlyD*, containing the three mutations in the *hlyB* gene, was verified *via* sequencing.

To introduce the mutations concerning the putative binding pocket outside (pbp-out), first plasmid pK184_*hly*B (V682)-*hly*D was constructed using primer sets of P5/P6, and then used as the backbone plasmid for the introduction of the second and third mutations. The PCR amplification of plasmid pK184_*hly*B (V682)-*hly*D was performed using the primer sets of P7/P8. The successful construction of plasmid pK184_*hly*B (V682D, L697D, Y700D)-*hly*D, containing three mutations in the *hly*B gene, was verified *via* sequencing.

### Mutations in the coding region of the nucleotide-binding domain of HlyB were introduced as follows

Plasmid pPSG122_*His*::*NBD* was used as the backbone plasmid. The *pBAD* promoter of this plasmid is inducible with L-arabinose. This plasmid has an ampicillin resistance gene. All the mutations in the *NBD* were introduced as described for the pK184_*hlyBD* backbone plasmid. The only exception was that primer P2’ was used instead of primer P2. Sequencing was performed to verify the successful construction of the desired plasmids, listed in Table 1.

### Expression and secretion experiments in shaker flasks

*Escherichia coli* BL21 (DE3) chemically competent cells were transformed with the desired plasmids and grown on LB agar plates supplemented with 50 μg/mL kanamycin and/or 100 μg/mL ampicillin.

In all of the cultures, first a mixture of transformed clones was used to prepare a pre-culture and cultivated overnight (37°C and 180 rpm). The overnight culture was used to inoculate 25 ml of 2YT (16 g/l Tryptone, 10 g/l yeast extract, 5 g/l NaCl) medium supplemented with 50 μg/mL kanamycin and/or 100 μg/mL ampicillin at an OD_600_ of 0.1 in 100 ml Erlenmeyer shaking flask. The cultures were cultivated at 37°C and 180 rpm to an OD_600_ of 0.7–0.8. Subsequently, the expression was induced with 1 mM IPTG and 5 mM CaCl_2_. Cells were grown for 4 h and samples were taken every hour. The samples were centrifuged (5,200 x g, 5 min). Then, supernatant and cell pellet were collected in separate Eppendorf tubes and mixed with an amount of SDS sample buffer to normalized the samples in the respect of the OD of cultures. Cell growth was also monitored each hour by measuring the OD_600_. The samples were analyzed either by SDS-PAGE or Western blot analysis. Staining of the SDS-PAGE gels was performed *via* the Colloidal Coomassie G-250 Staining protocol (Dyballa and Metzger, 2009). The intensity of the protein bands on the SDS-PAGE gels were semi-quantified using the ImageJ software (Image Processing and Analysis in Java) if necessary (Abràmoff et al., 2004).

To determine the amount of secreted target protein, a series of purified HlyA solutions with known concentrations were also loaded on the same SDS-PAGE. The amount of secreted target protein in the supernatant was then compared to the HlyA solution through a calibration line. The rate of secretion was calculated according to the published protocol (Lenders et al., 2016).

The supernatants samples were mixed with SDS-sample buffer. The pellet samples were first resuspended in resuspension buffer (50 mM Na_2_HPO_4_, pH 8, 300 mM NaCl) and then mixed with SDS-sample buffer. The supernatants and pellets samples were analyzed using SDS-PAGE analysis and Western-blotting.

### Expression and purification of HlyB nucleotide-binding domain variants

Expression and purification of the NBD variants were performed according to the published procedure (Zaitseva et al., 2004) with slight modifications, as described below.

The NBD plasmids (WT or mutant variants) were introduced into chemically competent *E. coli* BL21 (DE3) or *E. coli* BL21-Gold (DE3) cells. Then, the cells were grown on LB agar plates supplemented with 100 μg/mL ampicillin. A mixture of clones was used to prepare a pre-culture (50 ml 2YT) and cultivated overnight (37°C and 180 rpm). The overnight culture was used to inoculate 2 L of 2YT medium supplemented with 100 μg/mL ampicillin in a ratio of 1:100. The cultures were cultivated (37°C and 180 rpm) to an OD_600_ of 0.8. The cultures were cooled down to 20°C and cultured again to an OD_600_ of 1. Then, the cultures were induced with 0.002% L-arabinose and continued to grow for 3 h (20°C and 180 rpm). All following steps were performed at 4°C. Cells were harvested by centrifugation (20 min, 4,500 × g, 4°C). Cells were resuspended in buffer A (25 mM sodium phosphate, 100 mM potassium chloride, 10 mM imidazole, 20% Glycerol, pH 8) supplemented with protease inhibitor cocktails and DNAase. Cells were disrupted by passing three times through a cell disruptor (Microfluidizer M-110P, Microfluidics) at 1.5 kbar. Cell debris and undisrupted cells were removed by centrifugation (60 min, 125,000 × g, 4°C). The supernatant was loaded onto a 5 ml Zn^2+^-charged chelating HiTrap™ HP column that was already equilibrated with buffer A. The column washed with 25 ml of buffer A and the elution step was performed with a linear gradient of buffer A2 (25 mM sodium phosphate, 100 mM potassium chloride, 300 mM imidazole, 20% glycerol, pH 8). Based on SDS-PAGE analysis, fractions containing the HlyB NBD variants were mixed and concentrated to a final volume of 500 μL using an Amicon Ultra filter (15 ml, MWCO = 10,000 Da Merck/Milipore). The concentrated protein was centrifuged (10 min, 100,000 x g, 4°C) and subjected to a size-exclusion chromatography on the Superdex 200 increased 10/300 Gl column (GE Healthcare) in buffer B (10 mM CAPS-NaOH, 20% glycerol, pH 10.4). The purified HlyB NBD variants were concentrated using an Amicon Ultra filter (5 ml, MWCO = 10,000 Da Merck/Milipore), and used for further analysis.

### ATPase activity assays

ATPase assays were performed according to the published procedure (Zaitseva et al., 2005b) with slight modifications. In this assay, the amount of released free phosphate was determined *via* a colorimetric assay. The concentrated purified NBD was diluted in HEPES buffer (100 mM HEPES, 20% Glycerol, pH 7) only before starting the assay. Subsequently, 30 μL of NBD solution in HEPES buffer was added to 10 μL of 50 mM MgCl_2_ and 10 μL of ATP solution (to a final concentration of 0 to 6 mM, pH 8). Instead of MgCl_2,_ HEPES buffer was used in negative control samples. The ATPase assay was started by supplementing protein solution to the reaction.

The reaction mixture in a final volume of 50 μL was incubated for 120 min at 22° C. Then, 25 μL of sample reactions was added to a 96 well pellet and reaction stopped by adding 175 μL of 10 mM H_2_SO_4_. The amount of released free phosphate was monitored by adding 50 μL of staining solution (0.096% (w/v) malachite green, 1.48% (w/v) ammonium molybdate, 0.173% (w/v) Tween-20 in 2.36 M H_2_SO_4_). The mixture was incubated 8 min at room temperature. The amount of released free phosphate was recorded by measuring A_595 nm_ with a micro plate reader (iMark Microplate Reader, Bio Rad) and using phosphate buffer as standard. Raw data were fitted using GraphPad Prism 8 Software (GraphPad).

### Secondary structure prediction

Quick2D (Zimmermann et al., 2018) and AmphipaSeeK (Combet et al., 2000; Sapay et al., 2006) were used to predict the secondary structures. Quick2D is used for prediction of α-, π- and TM-helices, β-strands, coiled coils, as well as disordered regions (Zimmermann et al., 2018). AmphipaSeeK is specifically designed to identify amphipathic helices (Sapay et al., 2006). The AmphipaSeeK predicts a secondary structure, a membrane topology (in-plane or not-in-plane), a score for the proposed membrane topology, and an amphipathy score for each residue in dependence to the neighboring residues.

### Structure prediction of HlyB

The structure of HlyB was modeled based on the structure of PCAT1 with TopModel tool (Lin et al., 2015; Mulnaes et al., 2020). The monomers of our HlyB model superimposed with a RMSD of 3.3 Å (558 Cα atoms) and 3.6 Å (558 Cα atoms), respectively, on the monomers of the inward facing conformation of HlyB in the structure of the HlyB / HlyD complex (Zhao et al., 2022). These differences are due to a different orientation of TMD and NBD in our model compared to the single particle structure. Superimposition of the isolated TMDs revealed a RMSD of 1.8 Å (311 Cα atoms) and 1.3 Å (273 Cα atoms), respectively for the monomers of our HlyB model and the monomers of the inward facing conformation of HlyB in the HlyB / HlyD structure. The crystal structures of the isolated NBD of HlyB in the apo form (1MT0; Schmitt et al., 2003) and the ATP / Mg^2+^ bound state (1XEF; Zaitseva et al., 2005a) superimposed with RMSD values of 0.7 Å (191 Cα atoms, 1MT0) and 1.2 Å (449 Cα atoms, 1XEF), respectively. Due to the high structural identity between the crystal structures of the isolated NBD and the NBD in the single particle cryo-EM structure, we primarily used these crystal structures for our analysis.

### Illustration and visualization

We used Netwheel to visualize the amphipathic characteristics of a helix (Mol et al., 2018). Protein and peptide structures were processed in PyMOL (Delano, 2002; the PyMOL molecular Graphics System, Version 1.8.6.0 Enhanced for Mac OS X). In order to illustrate and identify hydrophobic surfaces the YRB-script was applied in PyMOL (Hagemans et al., 2015).

### Sequence alignments

Alignments were performed using Clustal Omega (Madeira et al., 2019). Sequences of the homologous proteins were taken from Uniprot^1^ or NCBI^2^.

## Results

### *In silico* studies on the putative HlyB binding pockets

It was proposed that in one of the early steps of the secretion, the secretion signal of HlyA interacts with the NBD of HlyB. Here, it was also reported that the presence of ATP accelerates the dissociation of this complex (Benabdelhak et al., 2003). Also, it was revealed that an amphipathic helix (AH) in the extreme C-terminus of HlyA plays an essential role in the secretion (Spitz et al., 2022). In this study, we assumed that the interaction between HlyA and NBD takes place within this essential AH. Based on this assumption, the binding region of the AH is expected to be a hydrophobic site. Accordingly, *in silico* studies explained in the following were performed.

It should be noted that different structures of the isolated NBD from different stages of ATP hydrolysis cycle are available, for instances: structures of the wild type NBD either as a nucleotide free monomer (PDB entry 1MT0; Benabdelhak et al., 2003), as an ADP bound monomer (PDB entry 2FF7; Zaitseva et al., 2005a), or as a TNP-ADP bound monomer (PDB entry 2PMK; Oswald et al., 2008). Additionally, the structures of the dimeric forms are available for the hydrolysis-impaired mutant E631Q with two ATP bound (PDB entry 2FGK; Zaitseva et al., 2005a), for the hydrolytic inactive mutant H662A (activity <0.1%; Zaitseva et al., 2005a) with two ATP bound (PDB entry 2FGJ; Zaitseva et al., 2005a), and for the mutant H662A with two ATP and two Mg^2+^ ions bound (PDB entry 1XEF; Zaitseva et al., 2005b). As outlined in Materials and Methods, the differences between the crystal structures of the isolated NBD of HlyB and the NBD of the HlyB structure in the cryo-EM structure of the HlyB / HlyD complex (Zhao et al., 2022) are marginal. Therefore, we focused on the structures of the isolated NBD as different conformations of the catalytic cycle are available.

Based on Benabdelhak et al., the interaction between HlyA and the NBD domain occurs in the absence of ATP, or on the other hand, the interaction occurs with the nucleotide free form of the NBD (Benabdelhak et al., 2003). Therefore, the structure of the nucleotide free monomer (PDB entry 1MT0) was used to search and identify putative hydrophobic binding pockets using the YRB-script (Hagemans et al., 2015). Furthermore, the dimeric ATP-bound structure (1XEF) was also analyzed to examine if the interaction is disrupted in the presence of ATP. Note that the positional changes of the residues can be analyzed by superimposing both structures and calculating the RMSD value only for the residues involved in forming the putative binding site. By performing this, we identified two regions of high hydrophobicity, which we called putative binding pocket inside (pbp-in) and putative binding pocket outside (pbp-out).

**Figure 1:**
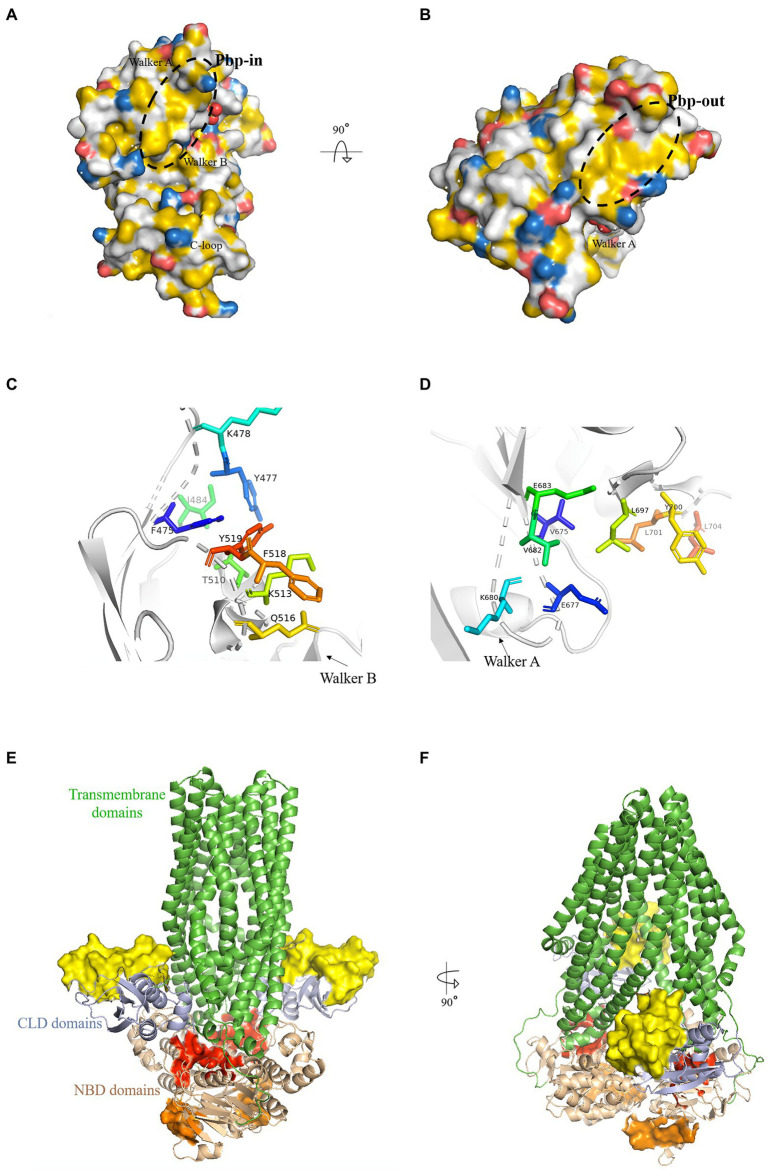
Putative binding pockets (pbp) of HlyB NBD. **(A,B)** The HlyB NBD monomer (1MT0) is shown in surface representation. The surface was colored with the YRB-script (Hagemans et al., 2015), which highlights carbon atoms that are not bound to oxygen or nitrogen in yellow, the charged oxygens of Glu and Asp in red, the charged nitrogen of Lys and Arg in blue while all other atoms in white. The structural elements of the NBD were labeled. The pbp’s are circled with a black dashed line. From panel **A**, which shows the pbp-in, to panel **B**, which shows the pbp-out, the molecules have been rotated toward the reader. **(C,D)** The residues of pbp-in **(C)** and pbp-out **(D)** from the monomeric structure (1MT0) are shown as stick representations. **(E,F)** Cartoon representation of a model of HlyB based on PCAT1 shown in green (Lin et al., 2015). The identified pbp’s are shown as surface representation with pbp-in in red and pbp-out in orange. The yellow surface maps the HlyA-interaction region on the CLD (Lecher et al., 2012). Pale colors correspond to the same regions in the second monomer. The overall domains of HlyB were labeled.

The pbp-in is located closely to the dimer interface and the ATP binding site (Figure 1A). While some residues from this pocket are polar amino acids, they contribute to the nonpolar character of the binding pocket with the carbon atoms of their side chains. Furthermore, charges at the side of a binding pocket for an AH may interact with the polar side of the AH and help its orientation. The residues that form pbp-in are: F475, Y477, K478, I484, T510, K513, Q516, F518, and Y519 (Figure 1C).

The pbp-in shares at least three residues with the ATP binding site (Y477, I484, T510): Y477, located in the A-loop (Ambudkar et al., 2006), interacts with the adenine base, I484 with the ribose moiety, and T510 with the Pα of ATP (Zaitseva et al., 2005a). When superimposing the ATP free monomer (1MT0) with the ATP bound dimer (1XEF) the residues of the pbp-in show a change in position of approximately 2 Å as reflected by the RMSD value. Both Tyr (Y477 and Y519) and both Lys (K487 and K513) display the largest change in position. The RMSD value is 1.3 Å when compared to the ADP-bound state (2FF7).

The pbp-out is located opposite to the membrane and is exposed to the cytosol (Figures 1B,F). It is made up by the following residues: V675, E677, K680, V682, E683, L697, Y700, L701 and L704 (Figure 1D). Y700, L701, and L704 point toward the dimer interface and are involved in monomer-monomer contacts (Zaitseva et al., 2005a) in the ATP-bound state. The pbp-out changes less than the pbp-in upon ATP binding and dimerization as shown by the RMSD value of 1.1 Å. The difference to the ADP-bound monomer is only 0.9 Å.

Both suggested pbp’s hold the potential to be the interaction sites of the C-terminal part of HlyA, as: (i) Both pbp’s display a hydrophobic region that matches the length of the AH in HlyA. (ii) They hold residues that are able to form π-π stacking interactions with their side chains and could, therefore, act as an interaction partner to F990 of HlyA. F990 is an essential amino acid of HlyA for the efficient secretion as revealed by mutagenesis studies (Chervaux and Holland, 1996). (iii) Both pbp’s are changed upon ATP binding and dimerization, supporting the already published observation of Benabdelhak et al. (2003).

### *In vivo* studies on the putative HlyB binding pockets

We applied directed mutagenesis to the proposed binding pockets to perform *in vivo* studies on the putative binding pockets. The first sets of mutagenesis experiments investigated the pbp-in. Two different variants of HlyB were cloned by introducing mutations at either two or three positions in the pbp-in site. One of the mutants harbored two point-mutations, F518D and Y519D, whereas the other one harbored three point-mutations, Y477D, F518D, and Y519D.

Additionally, the proposed pbp-out was investigated by mutagenesis studies. A pbp-out triple mutant was cloned that harbored the mutations V682D, L697D, and Y700D.

The constructed plasmids harboring different variants of HlyB protein were co-transformed into chemically competent *E. coli* BL21(DE3) cells along with pSU2627_*hlyC*(159 bp)-*hlyA* plasmid. Cell colonies harboring plasmids pK184_*hlyBD* and pSU2627_*hlyC*(159 bp)-*hlyA* were used as positive controls. Test expression of clones secreting HlyA was performed in shaking flasks employing directly the supernatant of cultures for analysis by SDS-PAGE. The test expression experiments were performed with three biological replicates.

**Figure 2:**
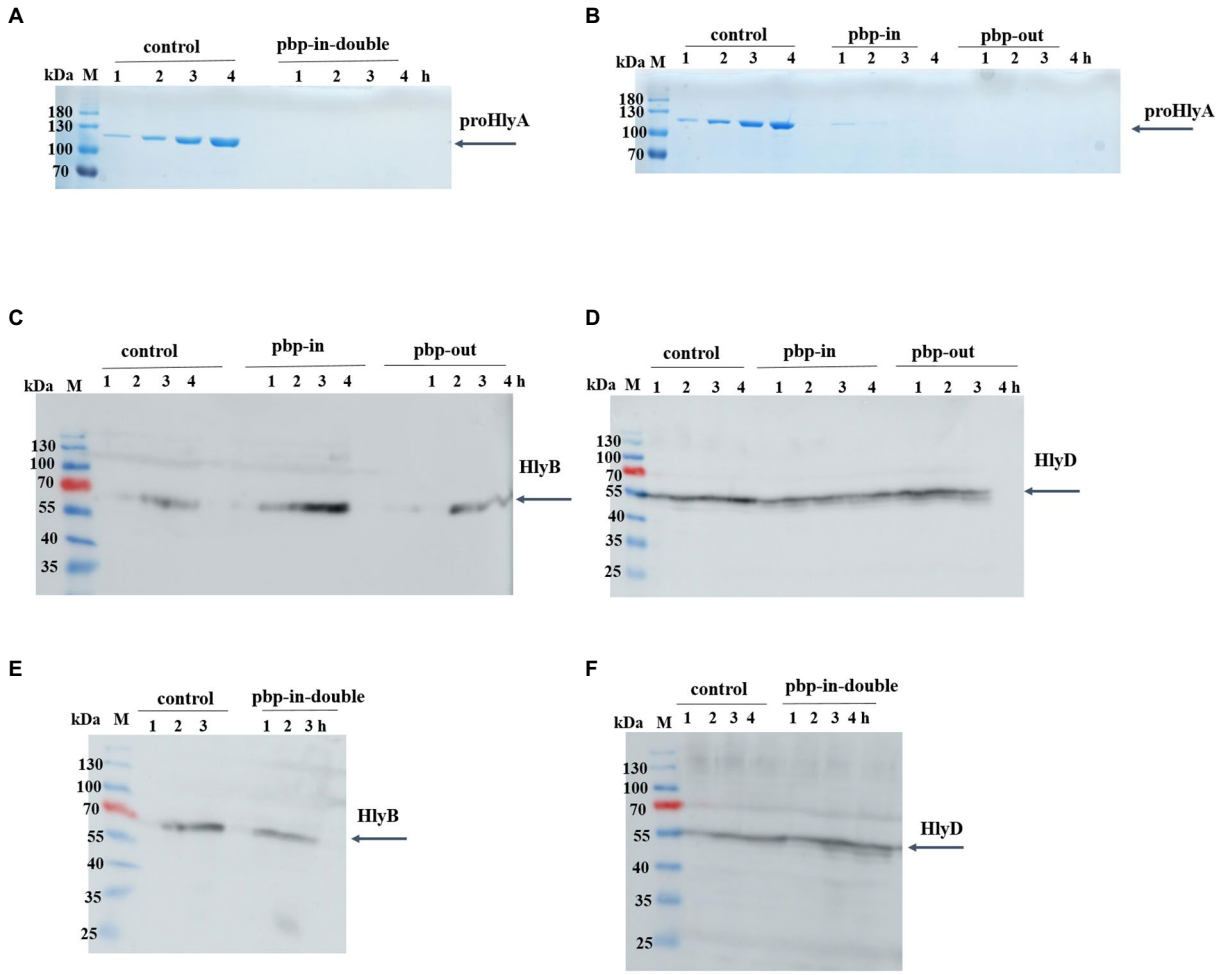
Secretion of proHlyA through the HlyA T1SS, in which HlyB harbors mutation. **(A)** SDS-PAGE analysis of the supernatant (unconcentrated) of clones secreting HlyA, in which HlyB is the wild type (control) or contains double mutations in pbp-in. **(B)** SDS-PAGE analysis of the supernatant (unconcentrated) of clones secreting HlyA, in which HlyB is the wild type (control), triple mutations in the pbp-in, or triple mutations in the pbp-out. Western blotting of *E. coli* cells (whole cells) demonstrated that **(C,E)** HlyB and **(D,F)** HlyD were expressed for all three mutants at levels comparable to the control cells. M, marker proteins; the molecular weight of the marker proteins is given on the left; xh, unconcentrated supernatant of culture, where x denotes the number of hours after induction.

Interestingly, we observed that neither the double pbp-in mutant (Figure 2A), triple pbp-in (Figure 2B), nor triple pbp-out mutant (Figure 2B) were able to secrete HlyA. However, in the same condition the positive controls were able to secrete HlyA (Figures 2A,B).

The expression level of HlyB and HlyD of the different mutants was analyzed using Western blotting demonstrating that HlyB (Figures 2C,E) and HlyD (Figures 2D,F) were successfully expressed in the different mutants. Thus, the lack of secretion of the mutants observed in our test secretion experiments cannot be attributed to the lack of the HlyA secretion system apparatus or individual components.

### *In vitro* studies on the putative HlyB binding pockets

**Figure 3:**
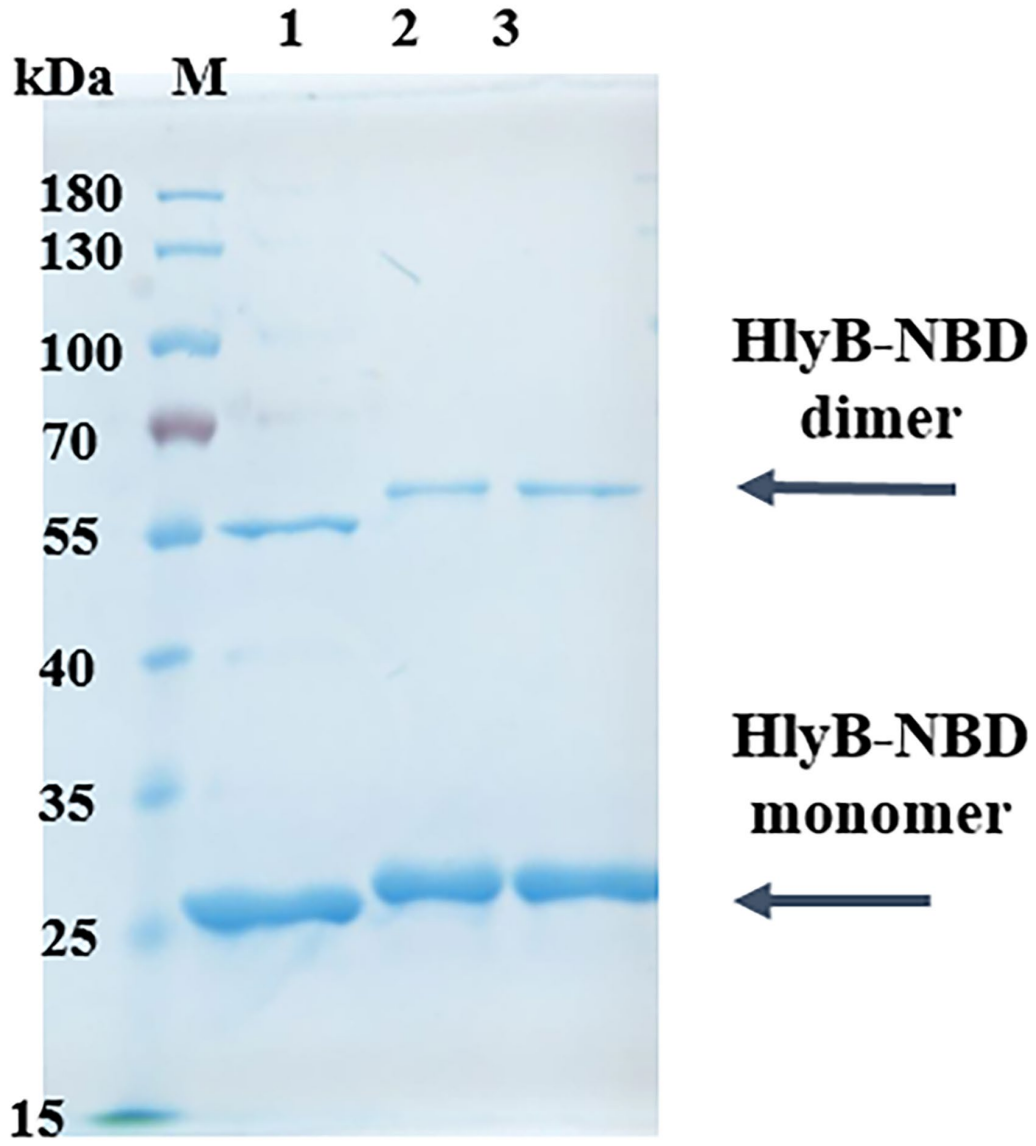
Purification of HlyB NBD mutants. SDS-PAGE of purified HlyB NBD of wild type (1), double-pbp-in (2), and (3) triple-pbp-in (3). M, marker proteins; the molecular weight of the marker proteins is given on the left.

To express and purify the HlyB-NBD mutants, a construct containing the C-terminal residues 467 to 707 of HlyB was overexpressed and purified according to the published protocol (Zaitseva et al., 2004). The expression and purification of wildtype HlyB-NBD, double-pbp-in mutant, and triple-pbp-in mutant were performed in high yield and purity (Figure 3). Gel shifting, which is uncorrelated migration with formula molecular weight of a protein on SDS-PAGE, was observed for the purified mutated NBDs (Figure 3). Amino acid substitutions can cause mobility changes during electrophoresis on SDS-PAGE. To date, several studies have investigated the correlation between the mobility changes and biochemical features of proteins, but still this relationship has to be explained (Rath et al., 2009; Shi et al., 2012). Shi et al. (2012) observed that net charge of a protein, binding ability to SDS-molecules are factors which affect protein migration on the SDS-PAGE.

Although, different expression strains and different expression conditions were tested, the purification of the pbp-out mutant was not successful.

### ATPase activity of the HlyB nucleotide-binding domain mutants

Isolated NBD variants were subsequently used to characterize the ATPase activity of each NBD. ATPase activity of the HlyB-NBD mutants was measured by a colorimetric assay based on the amount of released inorganic phosphate from the ATP hydrolysis.

The ATPase activity of the NBD pbp-in triple mutant was completely abolished (range of ATP concentrations 0 to 6 mM).

**Figure 4:**
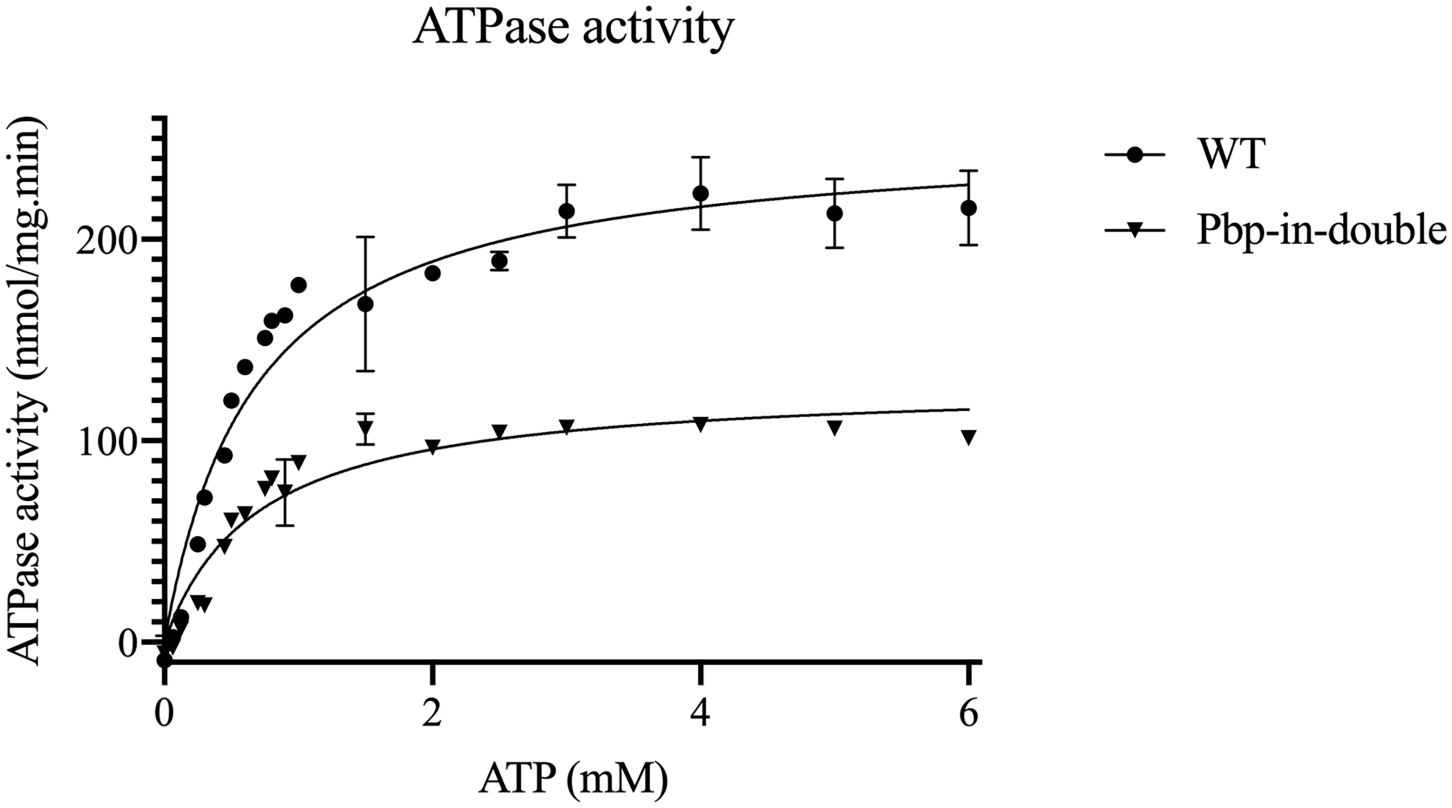
Kinetic measurements of the ATPase activity of HlyB NBD mutants, WT HlyB NBD (circle) and double-pbp-in (triangle). Data are from minimum two independent replicates, error bars were calculated by SEM.

The maximum velocity (V_max_) of 253.8 nmol/mg.min was determined for the wild type NBD under the conditions of the assay. The V_max_ of the NBD pbp-in double mutant was reduced two-fold in comparison to the wild type and determined to be 133.4 nmol/mg.min (Table 3; Figure 4). Despite of ATPase activity of the NBD pbp-in double mutant, no secretion was observed for this mutant.

**Table 3:** Summary of the kinetic parameters of HlyB NBD wild type and the mutated variants.

	Kinetic parameters	WT NBD	Pbp-in-double
Michaelis- Menten kinetic	V_max_ (nmol/mg.min)	253.8 ± 7.6	133.4 ± 7.9
	K_m_ (mM)	0.65 ± 0.06	0.73 ± 0.12
Hill equation	h	1.66 ± 0.154	2.37 ± 0.2
	K_0.5_ (1/min)	0.49 ± 0.026	0.5 ± 0.018

Note that the double and triple mutants of pbp-in differ in one residue (Y477), but the double mutant still is able to hydrolyze ATP, nevertheless, these mutations led to the lack of secretion. The kinetic parameters of the different ATPase activity are summarized in Table 3.

Y477 is a residue of the A-loop, which coordinates the adenine ring. Interestingly, Pdr5, the ABC transporter involved in pleiotropic resistance from baker’s yeast, have no aromatic A-loop residue but still able to hydrolyses ATP with high efficiency (Raschka et al., 2022).

### Investigations on the HlyB C39-peptidase-like domain

It is now understood that the CLD domain of HlyB is essential for secretion. Additionally, an interaction between HlyA and the CLD domain has been confirmed (Lecher et al., 2012). It seems that the AH at the C-terminus of HlyA has no impact on this interaction which was revealed by pull-down assay of the isolated CLD with unfolded/folded HlyA, HlyA1 (C-terminal 217 residues), and HlyA2 (HlyA1 lacking the C-terminal 60 residues that make up the secretion signal). Despite lacking the AH in HlyA2, no change in the interaction between the CLD and HlyA was observed. Instead, the interaction was disrupted when the experiment was performed with folded versions of the substrate (Lecher et al., 2012). In this regard, the authors assigned the interaction to the conserved GG repeats of the RTX domain of HlyA, which induce folding of RTX proteins upon Ca^2+^ binding (Baumann et al., 1993; Lecher et al., 2012).

By a combination of chemical shift experiments and mutational studies Lecher et al. were able to map the region in the CLD that interacts with HlyA (Figures 1E,F). Since HlyB seems to interact with different regions of the substrate, we were interested if a simultaneous binding would be possible.

**Figure 5:**
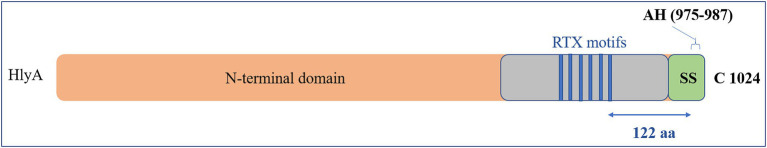
Schematic view of the HlyA and HlyA2. The distance the last C-terminal RTX and the beginning of the AH is 122 residues (122*3.8 Å = 463.6 Å).

The last C-terminal GG repeat of HlyA2 is located 122 amino acids upstream of the AH. Assuming 3.8 Å per amino acid in an unfolded protein (Carrion-Vazquez et al., 1999; Crecca and Roitberg, 2008), the distance between the last C-terminal GG repeat and the AH (NBD-interaction site) is approximately 450 Å. For a visualization of these distances see Figure 5.

In order to measure the distances between the pbp’s of the NBD and the interaction site on the CLD the structure of full-length HlyB is needed. However, the HlyB structure became available only recently and we modeled it based on another ABC transporters, PCAT1 (Lin et al., 2015; Figures 1E,F).

In this model the outmost residue of the HlyA interaction site in the CLD is E92. The minimal distance between E92 and pbp-in measures ~40 Å and the maximal distance from E92 to pbp-in is ~64 Å. The pbp-out shows a distance to E92 between 54 Å and 68 Å, respectively. Based on these calculations, HlyA can easily bridge these distances and simultaneous binding of the substrate HlyA to the CLD and NBD of HlyB is possible. In the recently published structure of the HlyB / HlyD complex (Zhao et al., 2022) only one of the three HlyB dimers is transport competent. Furthermore, only one of the two CLDs is visible in the electron density of the transport competent dimer. Here, the distance between E92 and pbp-in is approximately 60 Å, while the distance to pbp-out is with slight increased, approximately 75 Å. This also supports our conclusion that HlyA can bridge this distance and that the proposed simultaneous binding is indeed possible.

If multiple interaction sites on HlyB are occupied simultaneously by one substrate molecule, this would result in a strictly ordered substrate arrangement, which could confer specificity between substrate and transport machinery. The interaction between substrate and the secretion machinery has also essential impact on inducing the assembly of the translocation channel (Thanabalu et al., 1998; Zhao et al., 2022).

The transport components HlyB and HlyD can also secrete heterologously expressed RTX toxins such as FrpA from *N. meningitidis* and HlyIA from *A. pleuropneumoniae* serotype 1 (Gygi et al., 1990; Thompson and Sparling, 1993). Both are predicted to have an AH in their C-terminus and a GG repeat can be found 122 residues (FrpA) and 121 residues (HlyIA) upstream of this AH.

### Investigations on the conserved distance between the predicted amphipathic helix domain and the GG-repeat of HlyA

The secretion signal of HlyA is located in the C-terminal 60 residues (Holland et al., 2016). Interestingly, heterologous substrates that can be secreted by HlyBD show AHs in their C-terminal secretion signal when analyzed with the prediction tool AmphipaSeeK. Furthermore, these heterologous substrates display the same distance between the predicted AH and the GG-repeat of the RTX domain as HlyA (Table 4). A linker of a conserved length between the two interaction sites further strengthens the theory that the RTX domain and the secretion signal interact simultaneously with the ABC transporter, which might be important for the correct orientation of the substrate. Additionally, an aromatic residue (F990) close to the AH was shown to be important for HlyA secretion and a Phe residue close to the predicted AHs can be found in the heterologous substrates as well (Table 4).

**Table 4:** A list of heterologous proteins that can be secreted by the HlyA T1SS.

RTX protein	Host	Accession number	Uniprot entry link	Position of GG repeat	Position of AH	Distance GG-AH	Aromatic residue	Reference
HlyA	*E. coli*	P08715	https://www.uniprot.org/uniprotkb/P08715/entry	844	974	130	F990	
FrpA	*Neisseria meningitidis*	Q9K0K9	https://www.uniprot.org/uniprotkb/Q9K0K9/entry	1,125	1,257	132	F1274	Thompson and Sparling (1993)
FrpA	*Kingella kingae*	F5S7Z9	https://www.uniprot.org/uniprotkb/F5S7Z9/entry	600	720	120	F733	Erenburg (2022)
MbxA	*Mycobacterium bovis*	A7XER5	https://www.uniprot.org/uniprotkb/A7XER5/entry	756	886	130	F901	Erenburg (2022)
HlylA	*Actinobacillus pleuropneumoniae*	P55128	https://www.uniprot.org/uniprotkb/P55128/entry	840	969	129	F987	Gygi et al. (1990)
LktA	*Mannheimia haemolytica*	P0C083	https://www.uniprot.org/uniprotkb/P0C083/entry	779	909	130	F923	Highlander et al. (1990)
PaxA	*Pseudomonas aeruginosa*	Q9RCG8	https://www.uniprot.org/uniprotkb/Q9RCG8/entry	845	974	129	F991	Kuhnert et al. (2000)

**Figure 6:**
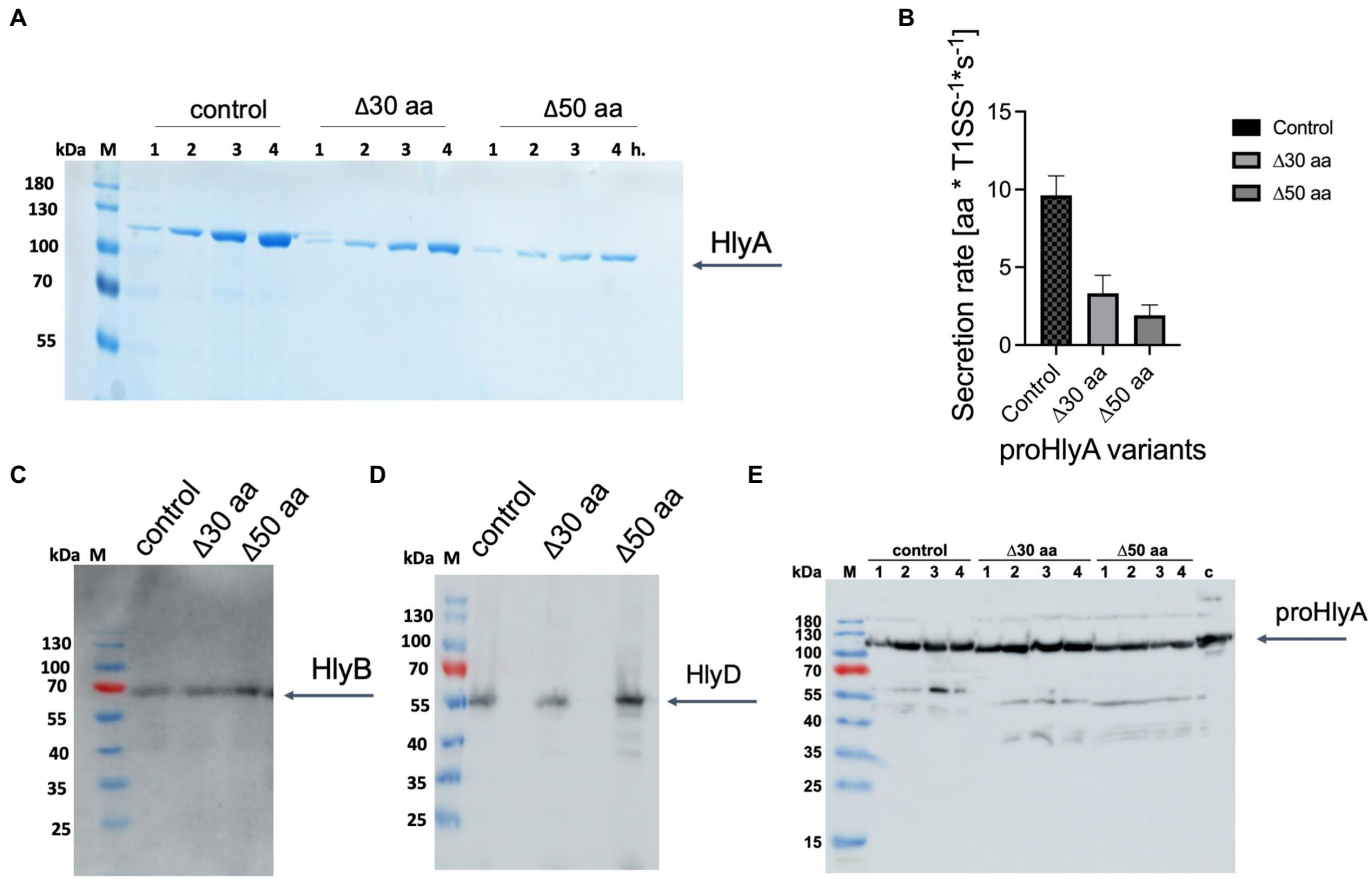
Secretion of proHlyA variants through the HlyA T1SS. **(A)** SDS-PAGE analysis of the supernatant (unconcentrated) of clones secreting wild type proHlyA (control), 30-residues-truncated proHlyA (Δ30 aa), and 50-residues-truncated proHlyA (Δ50 aa) through the HlyA T1SS. **(B)** Secretion rates of truncated proHlyA. Error bars represent the standard deviation of at least three biological replicates. Western blot analysis of *E. coli* cells demonstrated that the amounts of **(C)** HlyB, **(D)** HlyD or **(E)** proHlyA were expressed for cells secreting either WT proHlyA, 30-residues-truncated proHlyA, or 50-residues-truncated proHlyA in comparable levels. M, marker proteins; the molecular weight of the marker proteins is given on the left; x h., unconcentrated supernatant of culture, where x denotes the number of hours after induction.

The following mutagenesis experiments were performed to modify the length between the two docking sites, AH and GG repeats. Two different truncated versions of HlyA were cloned by deleting either 30 or 50 residues of the 130 residues that are encoded between the AH and the GG-repeats of HlyA. For the 30-residues-truncated HlyA, residues from 939 to 968 were deleted, and for the 50-residues-truncated HlyA, residues from 919 to 968 were deleted. The predicted AH domain is located between residues 975 to 987. The strain containing the plasmid encoding full-length HlyA was used as the control strain. Expression experiments of clones secreting wild type HlyA and both truncated variants of HlyA were performed in shaking flasks employing directly the supernatant of cultures for analysis by SDS-PAGE. The analysis confirmed that the secretion efficiency of both truncated HlyA variants were strongly reduced compared to the wild type HlyA (Figure 6A). The secretion rates of two HlyA variants were quantified according to the published protocol (Lenders et al., 2016). The secretion rates for the HlyA variants were displayed in Figure 6B indicating that the secretion rate of wild type HlyA is almost two-fold higher than the 30-residues-truncated HlyA variant. Additionally, the 50-residues-truncated HlyA variant showed the lowest secretion rate compared to the other two tested variants. Consequently, these observations demonstrated that by shortening the distance between the predicted AH domain and the GG-repeats of HlyA, the secretion level of this substrate is reduced.

The expression level of HlyB and HlyD of the different variants as well as the amount of HlyA was analyzed using Western blotting demonstrating that HlyB (Figure 6C), HlyD (Figure 6D), and pro HlyA (Figure 6E) were expressed in comparable levels in the different variants. Thus, the lower secretion of proHlyA observed in our test secretion experiments cannot be attributed to the lower amount of the secretion system apparatus or the secreted substrate.

## Discussion

The ABC transporter HlyB plays a central role in the HlyA T1SS, because it not only provides the energy of the transport by hydrolyzing ATP, but also plays a role in the early steps of the secretion by interacting with the substrate prior to the secretion. Previous studies have explored the relationship between HlyB and the substrate, and reported two interactions: An interaction site within the CLD domain (Lecher et al., 2012) and an interaction site within the NBD domain (Benabdelhak et al., 2003). Lecher et al. have been able to map the binding site of the CLD for HlyA (Lecher et al., 2012), but the binding site of the NBD for HlyA is still an open question. Here, we aimed to address this question by performing *in silico*, mutagenesis, and biochemical studies.

Recent studies demonstrated that the presence of an AH, covering the residues 970–987, in the C-terminus of HlyA is important for secretion. More important, this secondary structure rather than the primary structure plays a crucial role in the recognition of HlyA (Spitz et al., 2022). Furthermore, based on studies conducted by Benabdelhak et al. (2003), the interaction between the NBD and HlyA occurs within the C-terminus of the substrate, most probably the signal sequence is involved in this interaction, since HlyA2 lacking the C-terminal 57 amino acids does not interact with the NBD. Interestingly, the formed complex HlyB-NBD / C-terminus of HlyA dissociates when ATP is present (Benabdelhak et al., 2003).

Putting the pieces of this puzzle together, we assumed that the C-terminal AH is the site of HlyA that interacts with HlyB-NBD. To identify putative binding pocket(s) in HlyB, *in silico* studies were conducted and based on that, two putative binding pockets were identified, pbp-in and pbp-out. Interestingly, both binding pockets are located in hydrophobic regions with a size capable to accommodate the C-terminal AH of HlyA. Also, there are residues in both pockets that are able to form π-π interactions, thus those sites could be sites for binding of F990. This residue is in the C-terminus of HlyA, however, not part of the AH, but proven to be essential for secretion (Chervaux and Holland, 1996; Spitz et al., 2022). Both pockets showed changes upon ATP binding and dimerization that could go along with published observations (Benabdelhak et al., 2003) that ATP binding accelerates the dissociation of HlyB and HlyA.

A set of mutagenesis experiments were conducted on these putative binding pockets. First, for pbp-in two mutants were cloned, one mutant with three mutated residues (Y477D, F518D, and Y519D), and a mutant with two mutated residues (F518D, and Y519D). Since Y477 is a part of the ATP binding site, more precisely the A-loop, we decided for the variant with only two mutations, as well. For the pbp-out only a variant with three mutations was constructed (V682D, L697D, and Y700D).

Interestingly, neither of the variants were able to secrete HlyA anymore. It seems that HlyA cannot bind to the mutated binding sites, therefore, no secretion occurs. To examine ATPase activity of HlyB NBD variants, the corresponding mutants were generated, and purifications of all HlyB NBD variants were performed. Purification of the pbp-out NBD was not successful, although different expression strains and expression conditions were tested. The protein was in all cases expressed as inclusion bodies. This might indicate misfolding of the HlyB NBD resulting from the mutations in pbp-out.

Purification of the wild type NBD and both variants of pbp-in were performed in high yield and quality. It was not surprising that the pbp-in with three mutated residues showed no ATPase activity at all, because residue 477 is a part of the ATP binding site (A-loop) and mutation of this residue might affect the ATPase activity of the NBD. The results obtained from the ATPase activity experiment of pbp-in double mutations demonstrated an active NBD, despite lack of secretion for this mutant. What stands out from the secretion experiments and the ATPase activity assays, the pbp-in and pbp-out are two binding sites on NBD for HlyA. In other word, the results of *in silico*, *in vitro* and *in vivo* studies are in accordance with each other and support the proposal that the NBD of HlyB harbors two pbp’s in.

As discussed above, the second interaction between HlyB and HlyA occurs within the CLD. To assess if simultaneous HlyA binding to both areas, the NBD and the CLD, is possible, the distances between the domains known to interact with the C-terminal part of HlyA were determined in a model of the full-length ABC transporter HlyB. These measurements suggest that a simultaneous binding of the substrate to two domains of the transporter is possible.

Interestingly, heterologous substrates that can be secreted by HlyA T1SS, also show AHs in their C-terminal secretion signal when analyzed with the prediction tool AmphipaSeeK. These heterologous substrates display the same distance between the predicted AH and a GG-repeat of the RTX domain as HlyA. A linker of conserved length between the two interaction sites further strengthens the hypothesis that the RTX domain and the secretion signal interact simultaneously with the ABC transporter, which might be important for the correct orientation of the substrate. Additionally, an aromatic residue (F990) close to the AH was shown to be important for HlyA secretion and a Phe residue close to the predicted AHs can be found in the heterologous substrates as well. Further mutagenesis studies showed the importance of this consensus length, as shortening this distance significantly reduced the rate of secretion. Albeit the remarkable alteration of the conserved linker length (by deletion of 30 or 50 residues), still secretion of truncated HlyA occurred in a reasonable titers. This clearly demonstrates that a simultaneous binding of the two binding sites is not strictly necessary, although it significantly affects the efficiency of secretion and thus the amount of secreted HlyA.

## Conclusion

**Figure 7:**
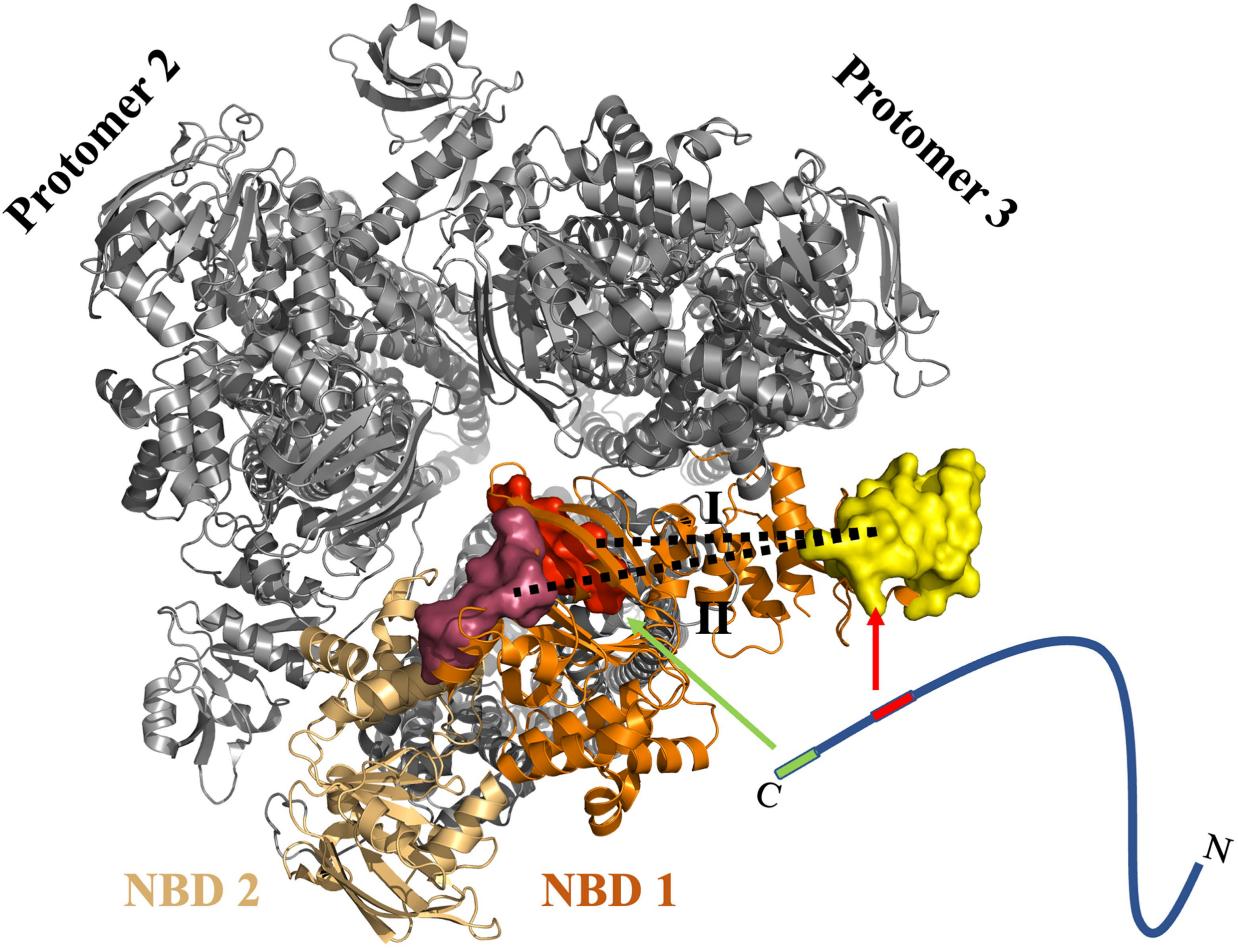
Proposed model of the interaction of HlyA with the NBD of HlyB. Cartoon representation of the trimer of dimers (PDB 7SGR) of the HlyB NBDs (Zhao et al., 2022). The view is from the cytosol toward the membrane. The NBDs of the transport-competent HlyB dimer are colored in orange and light orange, respectively, while the two non-transport competent HlyB dimers are shown in gray. The identified pbp’s are shown as surface representation with pbp-in in red and pbp-out in magenta. The yellow surface maps the HlyA-interaction region of the CLD. The substrate, HlyA, is drawn schematically as a line. The two interacting regions of HlyA are highlighted in green (secretion signal) and red (RTX domain). The arrows emphasis the interaction regions within the NBD of HlyB. Please note that the CLD of NBD 2 was not traced in the single particle cryo EM structure (Zhao et al., 2022). For most efficient secretion, HlyA has to interact simultaneously with both regions of the NBD. In the proposed model, a 1:1 stoichiometry of HlyA and HlyB is assumed. However, one cannot exclude a 2:1 stoichiometry as demonstrated for PCAT1 (Kieuvongngam et al., 2020).

This study demonstrated that NBD of the ABC transporter HlyB harbors two binding sites for the substrate. The presence of these two putative binding pockets could be validated by *in silico*, *in vitro*, and *in vivo* investigations. The modeling of the HlyB structure allowed measuring distances between the domains known to interact with HlyA’s C-terminal fragment. This measurement indicated that simultaneous binding of the substrate to two cytosolic domains (the CLD and the NBD) of the transporter is possible (Figure 7).

Last but not the least, we proposed that a consensus length between the GG repeats and the AH of the C-terminal HlyA is required for efficient secretion. The sequence analysis revealed that this consensus length is present in the heterologous RTX proteins secreted by the HlyA T1SS, while mutagenesis studies showed that shortening this distance reduces the secretion efficiency.

## Data availability statement

All data are avaliable upon request from the corresponding author.

## Author contributions

ZN: performing experiments and writing and editing the draft. EH: performing experiments and editing the draft. OS: performing experiments and writing the draft. SS: conceptualization and revising the draft for the content. LS: supervision, conceptualization, and revising the draft for the content. All authors contributed to the article and approved the submitted version.

## Funding

This research was funded by Deutsche Forschungsgemeinschaft through CRC 1208 project A01 to LS and in part by CLIB Competence Center Biotechnology (CKB) funded by the European Regional Development Fund ERDF (34.EFRE-0300096) to ZN and LS. The Center for Structural Studies was funded by the Deutsche Forschungsgemeinschaft (DFG Grant number 417919780) to SS.

## Conflict of interest

The authors declare that the research was conducted in the absence of any commercial or financial relationships that could be construed as a potential conflict of interest.

## Publisher’s note

All claims expressed in this article are solely those of the authors and do not necessarily represent those of their affiliated organizations, or those of the publisher, the editors and the reviewers. Any product that may be evaluated in this article, or claim that may be made by its manufacturer, is not guaranteed or endorsed by the publisher.

## Footnotes

^1^ https://www.uniprot.org/

^2^ https://www.ncbi.nlm.nih.gov/

## References

[ref1] Abràmoff M. D. Magalhães P. J. Ram S. J. (2004). Image processing with ImageJ. Biophoton. Int. 11, 36–42.

[ref2] Ambudkar S. V. Kim I.-W. Xia D. Sauna Z. E. (2006). The A-loop, a novel conserved aromatic acid subdomain upstream of the Walker a motif in ABC transporters, is critical for ATP binding. FEBS Lett. 580, 1049–1055. doi: 10.1016/j.febslet.2005.12.051, PMID: 16412422

[ref3] Baumann U. Wu S. Flaherty K. M. Mckay D. B. (1993). Three-dimensional structure of the alkaline protease of *Pseudomonas aeruginosa*: a two-domain protein with a calcium binding parallel beta roll motif. EMBO J. 12, 3357–3364. doi: 10.1002/j.1460-2075.1993.tb06009.x, PMID: 8253063 PMC413609

[ref4] Benabdelhak H. Kiontke S. Horn C. Ernst R. Blight M. A. Holland I. B.. (2003). A specific interaction between the NBD of the ABC-transporter HlyB and a C-terminal fragment of its transport substrate haemolysin a. J. Mol. Biol. 327, 1169–1179. doi: 10.1016/S0022-2836(03)00204-3, PMID: 12662939

[ref5] Bumba L. Masin J. Macek P. Wald T. Motlova L. Bibova I.. (2016). Calcium-driven folding of RTX domain β-rolls ratchets translocation of RTX proteins through type I secretion ducts. Mol. Cell 62, 47–62. doi: 10.1016/j.molcel.2016.03.018, PMID: 27058787

[ref6] Carrion-Vazquez M. Marszalek P. E. Oberhauser A. F. Fernandez J. M. (1999). Atomic force microscopy captures length phenotypes in single proteins. Proc. Natl. Acad. Sci. 96, 11288–11292. doi: 10.1073/pnas.96.20.11288, PMID: 10500169 PMC18026

[ref7] Chervaux C. Holland I. (1996). Random and directed mutagenesis to elucidate the functional importance of helix II and F-989 in the C-terminal secretion signal of *Escherichia coli* hemolysin. J. Bacteriol. 178, 1232–1236. doi: 10.1128/jb.178.4.1232-1236.1996, PMID: 8576066 PMC177793

[ref8] Combet C. Blanchet C. Geourjon C. Deleage G. (2000). NPS@: network protein sequence analysis. Trends Biochem. Sci. 25, 147–150. doi: 10.1016/S0968-0004(99)01540-6, PMID: 10694887

[ref9] Crecca C. R. Roitberg A. E. (2008). Using distances between α-carbons to predict protein structure. Int. J. Quantum Chem. 108, 2782–2792. doi: 10.1002/qua.21769

[ref10] Delano W. L. (2002). Pymol: an open-source molecular graphics tool. CCP4 Newsl. Prot. Crystall. 40, 82–92.

[ref11] Dyballa N. Metzger S. (2009). Fast and sensitive colloidal coomassie G-250 staining for proteins in polyacrylamide gels. JoVE e1431. doi: 10.3791/1431 PMC3149902 19684561

[ref12] Erenburg I. N. Hansch S. Chacko F. M. Hamacher A. Wintgens S. Stuhldreier F.. (2022). Heterologously secreted MbxA from Moraxella bovis induces a membrane blebbing response of the human host cell. Sci. Rep. 12:17825. doi: 10.1038/s41598-022-22480-x 36280777 PMC9592583

[ref13] Gygi D. Nicolet J. Frey J. Cross M. Koronakis V. Hughes C. (1990). Isolation of the Actinobacillus pleuropneumoniae haemolysin gene and the activation and secretion of the prohaemolysin by the HlyC, HlyB and HlyD proteins of *Escherichia coli*. Mol. Microbiol. 4, 123–128. doi: 10.1111/j.1365-2958.1990.tb02021.x, PMID: 2181233

[ref14] Hagemans D. Van Belzen I. A. Morán Luengo T. Rüdiger S. G. (2015). A script to highlight hydrophobicity and charge on protein surfaces. Front. Mol. Biosci. 2:56. doi: 10.3389/fmolb.2015.00056 26528483 PMC4602141

[ref15] Hardie K. Issartel J. P. Koronakis E. Hughes C. Koronakis V. (1991). In vitro activation of *Escherichia coli* prohaemolysin to the mature membrane-targeted toxin requires HlyC and a low molecular-weight cytosolic polypeptide. Mol. Microbiol. 5, 1669–1679. doi: 10.1111/j.1365-2958.1991.tb01914.x, PMID: 1943702

[ref16] Highlander S. K. Engler M. J. Weinstock G. M. (1990). Secretion and expression of the Pasteurella haemolytica leukotoxin. J. Bacteriol. 172, 2343–2350. doi: 10.1128/jb.172.5.2343-2350.1990, PMID: 2185213 PMC208868

[ref17] Holland I. B. Peherstorfer S. Kanonenberg K. Lenders M. Reimann S. Schmitt L. (2016). Type I protein secretion-deceptively simple yet with a wide range of mechanistic variability across the family. EcoSal Plus 7, 1–46. doi: 10.1128/ecosalplus.ESP-0019-2015, PMID: 28084193 PMC11575716

[ref18] Khosa S. Scholz R. Schwarz C. Trilling M. Hengel H. Jaeger K.-E.. (2018). An a/U-rich enhancer region is required for high-level protein secretion through the hlya type i secretion system. Appl. Environ. Microbiol. 84, e01163–e01117. doi: 10.1128/AEM.01163-17 PMC5734041 29030442

[ref19] Kieuvongngam V. Olinares P. D. B. Palillo A. Oldham M. L. Chait B. T. Chen J. (2020). Structural basis of substrate recognition by a polypeptide processing and secretion transporter. elife 9:e51492. doi: 10.7554/eLife.51492, PMID: 31934861 PMC6959990

[ref20] Koronakis V. Koronakis E. Hughes C. (1989). Isolation and analysis of the C-terminal signal directing export of *Escherichia coli* hemolysin protein across both bacterial membranes. EMBO J. 8, 595–605. doi: 10.1002/j.1460-2075.1989.tb03414.x, PMID: 2656259 PMC400846

[ref21] Kuhnert P. Heyberger-Meyer B. Nicolet J. Frey J. (2000). Characterization of PaxA and its operon: a cohemolytic RTX toxin determinant from pathogenic Pasteurella aerogenes. Infect. Immun. 68, 6–12. doi: 10.1128/IAI.68.1.6-12.2000, PMID: 10603361 PMC97094

[ref22] Lecher J. Schwarz C. K. Stoldt M. Smits S. H. Willbold D. Schmitt L. (2012). An RTX transporter tethers its unfolded substrate during secretion via a unique N-terminal domain. Structure 20, 1778–1787. doi: 10.1016/j.str.2012.08.005, PMID: 22959622

[ref23] Lenders M. H. Beer T. Smits S. H. Schmitt L. (2016). In vivo quantification of the secretion rates of the hemolysin a type I secretion system. Sci. Rep. 6:33275. doi: 10.1038/srep33275, PMID: 27616645 PMC5018854

[ref24] Lenders M. H. Weidtkamp-Peters S. Kleinschrodt D. Jaeger K.-E. Smits S. H. Schmitt L. (2015). Directionality of substrate translocation of the hemolysin a type I secretion system. Sci. Rep. 5:12470. doi: 10.1038/srep12470, PMID: 26212107 PMC4648471

[ref25] Letoffe S. Delepelaire P. Wandersman C. (1996). Protein secretion in gram-negative bacteria: assembly of the three components of ABC protein-mediated exporters is ordered and promoted by substrate binding. EMBO J. 15, 5804–5811. doi: 10.1002/j.1460-2075.1996.tb00967.x, PMID: 8918458 PMC452328

[ref26] Lin D. Y.-W. Huang S. Chen J. (2015). Crystal structures of a polypeptide processing and secretion transporter. Nature 523, 425–430. doi: 10.1038/nature14623, PMID: 26201595

[ref27] Linhartová I. Bumba L. Mašín J. Basler M. Osička R. Kamanová J.. (2010). RTX proteins: a highly diverse family secreted by a common mechanism. FEMS Microbiol. Rev. 34, 1076–1112. doi: 10.1111/j.1574-6976.2010.00231.x, PMID: 20528947 PMC3034196

[ref28] Madeira F. Park Y. M. Lee J. Buso N. Gur T. Madhusoodanan N.. (2019). The EMBL-EBI search and sequence analysis tools APIs in 2019. Nucleic Acids Res. 47, W636–W641. doi: 10.1093/nar/gkz268, PMID: 30976793 PMC6602479

[ref29] Mol A. R. Castro M. S. Fontes W. (2018). NetWheels: a web application to create high quality peptide helical wheel and net projections. BioRxiv 416347. doi: 10.1101/416347

[ref30] Mulnaes D. Porta N. Clemens R. Apanasenko I. Reiners J. Gremer L.. (2020). TopModel: template-based protein structure prediction at low sequence identity using top-down consensus and deep neural networks. J. Chem. Theory Comput. 16, 1953–1967. doi: 10.1021/acs.jctc.9b00825, PMID: 31967823

[ref31] Nicaud J.-M. Mackman N. Gray L. Holland I. (1986). The C-terminal, 23 kDa peptide of *E. coli* haemolysin 2001 contains all the information necessary for its secretion by the haemolysin (Hly) export machinery. FEBS Lett. 204, 331–335. doi: 10.1016/0014-5793(86)80838-9, PMID: 3525227

[ref32] Noegel A. Rdest U. Springer W. Goebel W. (1979). Plasmid cistrons controlling synthesis and excretion of the exotoxin α-haemolysin of *Escherichia coli*. Mol. Gen. Genet. MGG 175, 343–350. doi: 10.1007/BF00397234, PMID: 392234

[ref33] Oswald C. Holland I. B. Schmitt L. (2006). The motor domains of ABC-transporters. Naunyn Schmiedeberg's Arch. Pharmacol. 372, 385–399. doi: 10.1007/s00210-005-0031-4, PMID: 16541253

[ref34] Oswald C. Jenewein S. Smits S. H. Holland I. B. Schmitt L. (2008). Water-mediated protein-fluorophore interactions modulate the affinity of an ABC-ATPase/TNP-ADP complex. J. Struct. Biol. 162, 85–93. doi: 10.1016/j.jsb.2007.11.006, PMID: 18155559

[ref35] Pourhassan N. Z. Cui H. Khosa S. Davari M. D. Jaeger K. E. Smits S. H.. (2022). Optimized Hemolysin type 1 secretion system in *Escherichia coli* by directed evolution of the Hly enhancer fragment and including a terminator region. Chembiochem 23:e202100702. doi: 10.1002/cbic.202100702 35062047 PMC9306574

[ref36] Pourhassan Z. Smits S. H. Ahn J. H. Schmitt L. (2021). Biotechnological applications of type 1 secretion systems. Biotechnol. Adv. 53:107864. doi: 10.1016/j.biotechadv.2021.107864, PMID: 34767962

[ref37] Raschka S. L. Harris A. Luisi B. F. Schmitt L. (2022). Flipping and other astonishing transporter dance moves in fungal drug resistance. BioEssays 44:2200035. doi: 10.1002/bies.202200035 35451123

[ref38] Rath A. Glibowicka M. Nadeau V. G. Chen G. Deber C. M. (2009). Detergent binding explains anomalous SDS-PAGE migration of membrane proteins. Proc. Natl. Acad. Sci. 106, 1760–1765. doi: 10.1073/pnas.0813167106, PMID: 19181854 PMC2644111

[ref39] Sapay N. Guermeur Y. Deléage G. (2006). Prediction of amphipathic in-plane membrane anchors in monotopic proteins using a SVM classifier. BMC Bioinformatics 7, 1–11. doi: 10.1186/1471-2105-7-255 16704727 PMC1564421

[ref40] Schmitt L. Benabdelhak H. Blight M. A. Holland I. B. Stubbs M. T. (2003). Crystal structure of the nucleotide-binding domain of the ABC-transporter haemolysin B: identification of a variable region within ABC helical domains. J. Mol. Biol. 330, 333–342. doi: 10.1016/S0022-2836(03)00592-8, PMID: 12823972

[ref41] Shi Y. Mowery R. A. Ashley J. Hentz M. Ramirez A. J. Bilgicer B.. (2012). Abnormal SDS-PAGE migration of cytosolic proteins can identify domains and mechanisms that control surfactant binding. Protein Sci. 21, 1197–1209. doi: 10.1002/pro.2107, PMID: 22692797 PMC3537240

[ref42] Spitz O. Erenburg I. N. Kanonenberg K. Peherstorfer S. Lenders M. H. Reiners J.. (2022). Identity determinants of the translocation signal for a type 1 secretion system. Front. Physiol. 12:2585–2598. doi: 10.3389/fphys.2021.804646 PMC8870123 35222063

[ref43] Thanabalu T. Koronakis E. Hughes C. Koronakis V. (1998). Substrate-induced assembly of a contiguous channel for protein export from E. coli: reversible bridging of an inner-membrane translocase to an outer membrane exit pore. EMBO J. 17, 6487–6496. doi: 10.1093/emboj/17.22.6487, PMID: 9822594 PMC1170996

[ref44] Thomas S. Holland I. B. Schmitt L. (2014). The type 1 secretion pathway—the hemolysin system and beyond. Biochimica et Biophysica Acta (BBA)-molecular. Cell Res. 1843, 1629–1641. doi: 10.1016/j.bbamcr.2013.09.017 24129268

[ref45] Thompson S. A. Sparling P. (1993). The RTX cytotoxin-related FrpA protein of *Neisseria meningitidis* is secreted extracellularly by meningococci and by HlyBD+ *Escherichia coli*. Infect. Immun. 61, 2906–2911. doi: 10.1128/iai.61.7.2906-2911.1993, PMID: 8514394 PMC280938

[ref46] Zaitseva J. Holland I. B. Schmitt L. (2004). The role of CAPS buffer in expanding the crystallization space of the nucleotide-binding domain of the ABC transporter haemolysin B from *Escherichia coli*. Acta Crystallogr. D Biol. Crystallogr. 60, 1076–1084. doi: 10.1107/S0907444904007437 15159567

[ref47] Zaitseva J. Jenewein S. Jumpertz T. Holland I. B. Schmitt L. (2005a). H662 is the linchpin of ATP hydrolysis in the nucleotide-binding domain of the ABC transporter HlyB. EMBO J. 24, 1901–1910. doi: 10.1038/sj.emboj.7600657, PMID: 15889153 PMC1142601

[ref48] Zaitseva J. Jenewein S. Wiedenmann A. Benabdelhak H. Holland I. B. Schmitt L. (2005b). Functional characterization and ATP-induced dimerization of the isolated ABC-domain of the haemolysin B transporter. Biochemistry 44, 9680–9690. doi: 10.1021/bi0506122, PMID: 16008353

[ref49] Zhao H. Lee J. Chen J. (2022). The hemolysin a secretion system is a multi-engine pump containing three ABC transporters. Cells 185, 3329–3340 e13. doi: 10.1016/j.cell.2022.07.017 36055198

[ref50] Zimmermann L. Stephens A. Nam S.-Z. Rau D. Kübler J. Lozajic M.. (2018). A completely reimplemented MPI bioinformatics toolkit with a new HHpred server at its core. J. Mol. Biol. 430, 2237–2243. doi: 10.1016/j.jmb.2017.12.007, PMID: 29258817

